# A New Temporary Training Prosthesis for People With Transtibial Amputation: a Technical Note

**DOI:** 10.33137/cpoj.v7i1.43034

**Published:** 2024-06-30

**Authors:** H Gholizadeh, N Baddour, N Dudek, E.D. Lemaire

**Affiliations:** 1 Department of Mechanical Engineering, University of Ottawa, Ottawa, Canada.; 2 AMPOS Orthopaedics Inc, Ottawa, Canada.; 3 Department of Medicine, (Division of Physical Medicine & Rehabilitation) and The Ottawa Hospital, University of Ottawa, Canad; 4 Department of Medicine, Faculty of Medicine, University of Ottawa, Ottawa, Canada; 5 Centre for Rehabilitation Research and Development, Ottawa Hospital Research Institute, Ottawa, Canada

**Keywords:** Amputation, Training Prosthesis, Artificial Limb, Post-Operative Prosthesis, Prosthetic Users, Rehabilitation, Quality of Life, Below Knee Amputation, Temporary Prosthesis

## Abstract

**BACKGROUND:**

While waiting to receive a prosthesis, individuals with amputations could benefit from using a temporary training prosthesis to expedite the rehabilitation process and prepare them for subsequent walking with their prosthesis.

**OBJECTIVES:**

To design and build a temporary training prosthesis for people with a transtibial amputation.

**METHODOLOGY:**

Various temporary training prostheses were designed and simulated using SolidWorks software, followed by fabricating and testing multiple prototypes. Initial tests were conducted on five able bodied subjects without amputation to evaluate comfort, ensure the prototype functioned as intended, and to refine the design. The final prototype design had no weight-bearing on the residual limb end and required the person to wear a shrinker or silicone liner.

**FINDINGS:**

SolidWorks simulations showed that the device could tolerate up to 200 kg load. Subjective feedback indicated that body weight is primarily supported by the thigh section, while partially utilizing the patellar tendon and tibial flares. The thigh section can be shifted 5 cm up or down and 2.5 cm to the front or back from the knee joint center (to enhance knee stability or function). Additionally, the thigh angle can be adjusted to 0, 5, 10, or 15 degrees to accommodate hip flexion contracture. The shank section width is adjustable and can be shifted up or down based on the residual limb shape. All five able-bodied participants successfully walked with the non-amputee version of the temporary prosthesis prototype and the device withstood walking, sitting, and standing loads.

**CONCLUSION:**

An adjustable temporary training prosthesis was successfully designed, and pilot tested by five able-bodied individuals. Future testing will involve five experienced prosthetic users before conducting trials with individuals with a new transtibial amputation.

## INTRODUCTION

Limb amputation is a meaningful event that can have both a physical and psychological impact on an individual’s well-being. After lower limb amputation, comprehensive rehabilitation is essential for regaining independence, mobility, and enhancing quality of life. Before receiving a prosthesis, people with amputation often rely only on wheelchairs or crutches for mobility.^1,2^

Prosthetic fitting typically commences between one to six months after amputation once swelling has subsided and the surgical incision has fully healed. During this phase, while the person is awaiting their prosthesis, a temporary prosthesis may facilitate early ambulation and enable individuals to initiate walking post-amputation.^1–3^ People with lower limb amputation can utilize a temporary prosthesis within parallel bars or with the assistance of crutches or a cane to enhance residual limb strength and endurance. Temporary prostheses or post operative prosthesis can play an important role in the overall success of rehabilitation for people with lower limb amputation, contributing to the prevention of complications associated with prolonged bed rest and promoting early discharge from the hospital.^**2,4–6**^

Very few types of temporary or post operative prostheses are available on the market. In post-amputation rehabilitation, the Pneumatic Post-Amputation Mobility Aid (PPAM) is a device designed to improve balance and gait in individuals with a new lower limb amputation and aid in the reduction of residual limb edema, thereby preparing the residual limb for wearing a prosthesis. However, the device is very bulky, and donning and doffing is not convenient for patients because they require assistance from clinicians, cannot manage the device independently, and cannot use it at home. Moreover, patients cannot bend their knee and gait is not symmetrical.^7^

Immediate post-operative rigid dressings with a pylon/foot fitting are also employed in rehabilitation. The concept of rigid dressings was first introduced by Muirhead Little during the First World War.^2^ Immediate post-operative rigid dressings can promote primary wound healing, facilitate stump shrinkage, prevent knee contracture, and enable early patient mobilization.^1,4–6^ However, by immobilizing the knee, the rigid dressing obstructs knee joint rehabilitation, resulting in unnatural gait that contradicts rehabilitation goals.^8^

The Bent-Knee Temporary Prosthesis (BKTP) is another device designed to pad and offload the residual limb.^9^ However, a primary concern with BKTP and similar devices like the iWalk^10^ is that the knee is positioned in ninetydegree flexion, which can lead to knee contracture and asymmetric gait. Prolonged use of devices like the iWalk can indeed result in significant changes to an individual’s gait, causing asymmetrical walking.^10^

A simple-to-use, temporary training prosthesis could assist individuals with transtibial amputation in learning how to walk with a prosthesis and remaining active while awaiting to receive their prosthesis. The device should not restrict the knee joint and should be easy to don and doff. Additionally, weight distribution between the residual limb and thigh section is crucial to alleviate pressure on the newly amputated leg. Therefore, the objective of this research was to develop a new temporary training prosthesis for individuals with new transtibial amputations.

## METHODOLOGY

A temporary training prosthesis model was designed and simulated using SolidWorks software (version 2020). The prototype design criteria were based on the research team's clinical experiences working with people with amputation over many years, as well as on a thorough literature review and discussions with experts. The main criteria were:

- The device should allow a knee range of motion of at least 120°.
- Thigh section angle should be adjustable within a range of 15°.
- The device should accommodate different thigh lengths by allowing for a 5 cm shift up or down in the thigh section, resulting in a total adjustment of 10 cm.
- The device should alleviate pressure on the newly amputated leg. Users should be able to stand and put weight on the device using only the thigh section (i.e., shank section is open).
- The device is easy to don and doff. Users should be able to put on and take off the prosthesis within 3 minutes.
- The device should be easy to adjust for clinicians. Adjustments should be possible using standard tools available in most clinical settings, with no more than 2 different tools required and taking a maximum of 15 minutes to adjust for different patients.
- The device should weigh less than 4 kg, similar to a transtibial prosthesis with a thigh shell.

**Figure 1:**
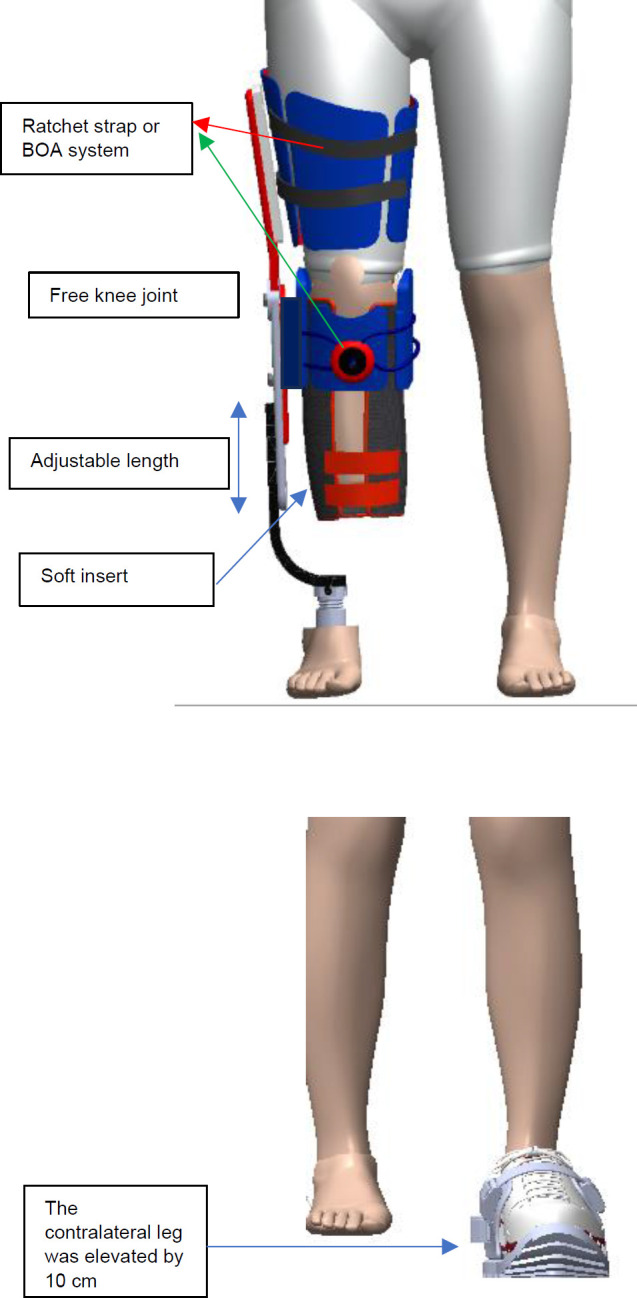
**Top**: Initial design concept for a temporary training prosthesis for people with transtibial amputation; **Bottom**: The contralateral leg was elevated by 10 cm to provide sufficient room for the other leg to suspend inside the device.

Based on these criteria, a design was developed featuring a single upright, with the thigh and shank sections unilaterally connecting to the knee joint and prosthetic foot (**Figure 1**). The first prototype featured thigh and shank sections made of polypropylene, with the connection between the upright and the prosthetic foot made of carbon fiber, fiberglass, and aluminium.

To evaluate comfort and function before testing on individuals with amputations, and to determine whether design criteria/objectives have been met, this temporary prosthesis was adapted for individuals without amputations. This approach allowed both the research team and able-bodied individuals to experience using the device and provide subjective feedback to improve the design. Therefore, the shank section was made longer to match different leg lengths and to add 1 cm clearance between the distal end of the foot and the device. Additionally, the contralateral leg was elevated by 10 cm using a portable shoe sole made of ethylene vinyl acetate (EVA) foam, which can be used with any type of shoe. This configuration ensured that only one intact limb and the contralateral temporary training prosthesis made contact with the ground (**Figure 1**).

The research prosthetist wore the first prototype inside a prosthetic facility, using parallel bars, and provided feedback on various aspects such as comfort during use, the ability to adjust the prototype for optimal fit, ease of donning and doffing the prototype, and evaluations of stability and functionality.

The thigh and shank could be adjusted using a ratchet strap or a BOA system (https://www.boafit.com/en-us/bracing). The knee was free, and the length of the device was adjustable. In the initial prototype, the thigh angle could not be adjusted to accommodate hip contracture. Due to the flexible nature of the thigh and shank sections, the user could not bear body weight adequately, resulting in the thigh and shank shells, shifting downward medially, where no upright support was present, by approximately 6 cm during weight-bearing.

**Figure 2:**
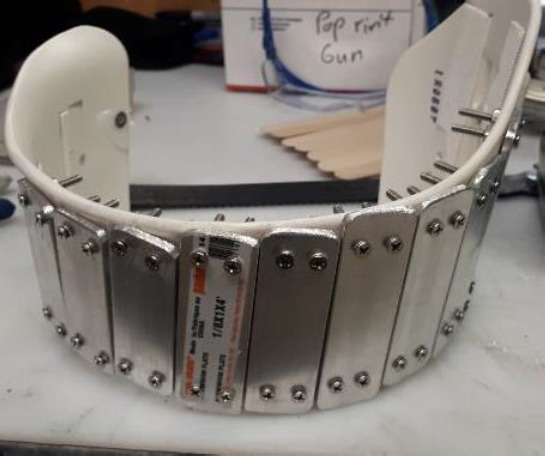
Watch band concept for the thigh shell.

One solution was to increase the strength of the thigh and shank sections (for example, using carbon fiber) to decrease or eliminate vertical movement during weight bearing. However, this would reduce shell flexibility, which was necessary to adjust for different thigh or shank sizes. Several ideas were considered to maintain shell flexibility while controlling vertical movement during weight bearing. In one design (**Figure 2**) a watch band concept was used to make the shells flexible in the horizontal plane while keeping them rigid in the vertical direction. However, after trying different prototypes, the team was unable to completely control vertical movement (i.e., there was still 1 cm movement).

Based on the issues encountered with the first prototype and prosthetist feedback, the research team modified the design by incorporating two uprights, one medial and one lateral, to better control movement in the shells, and added an adapter for adjusting the thigh section angle.

**Figure 3:**
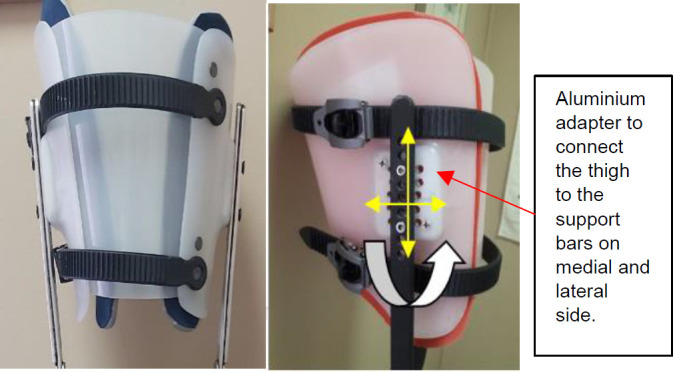
Thigh shell with aluminium adapter. **Left**: front view with vinyl cover; **Right**: lateral view.

Throughout this iterative process, the design was refined to ensure adaptability to a broader population and meeting the specific needs of people with a transtibial amputation. The final prototype design includes two aluminium adapters (4.5 cm height, 3.6 cm length, 0.5 cm width) to connect the thigh to the support bars (uprights) on medial and lateral side. This adapter has unique threaded hole arrangements to allow for front/back shifting, up/down movement, and flexion adjustment, (**Figure 3**). Multiple iterations of this adapter were developed and tested clinically to ensure adequate adjustability to meet diverse patient needs.

The thigh shell was fabricated from polypropylene (5 mm sheet), with 1 cm Plastazote^®^ foam padding inside the shell for comfort. Additionally, a vinyl cover was applied to areas that contact the leg to facilitate easy cleaning (**Figure 3**).

Two off-the-shelf locking knee joints (Modified Ring Lock Knee Joint) from Becker Orthopedic were used (https://beckerorthopedic.com/Product/KneeJoints/1000KneeSeries/1002#selection-guide) to provide unrestricted knee motion during ambulation, with the option for the patient to lock the knee joint if necessary. To connect the joint bars to the designed adapter, several holes (one centimetre apart) were drilled into the bars to accommodate M6 screws. SolidWorks software simulation (using forces for a person weighing 100 kg) indicated the need for increased durability. Therefore, 8 mm thick medial and lateral aluminum bars were riveted to the joint bars using 3 mm stainless steel rivets.

**Figure 4:**
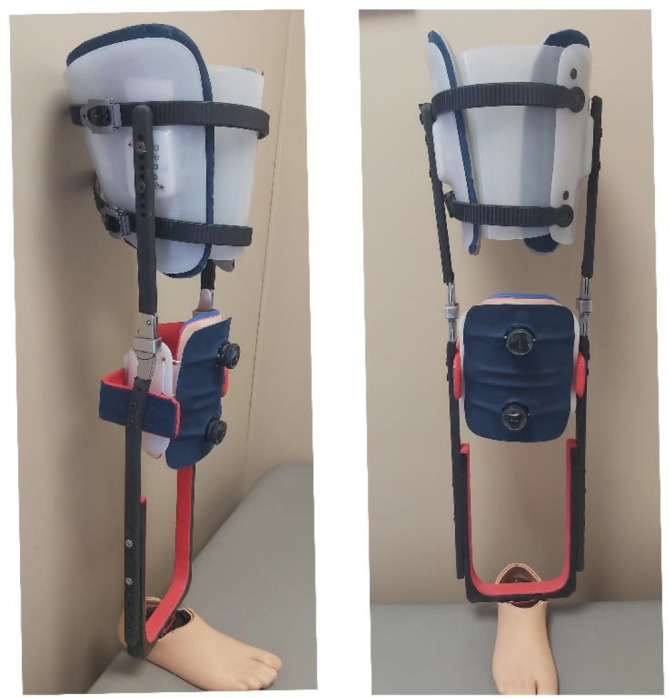
The temporary training prosthesis adapted for able-bodied individuals. This version is longer in the shank section to provide sufficient room for the leg to suspend inside the device.

In the shank section, sufficient room between the bars is needed to accommodate swollen and/or larger legs. Therefore, the width must be adjustable to accommodate different shank sizes. The width is adjustable in the shank section using four M6 screws, and the shank section can also be shifted up or down (**Figure 4**).

After receiving approval from the University of Ottawa Research Ethics Board (H-09-22-8410), initial testing was conducted with five male individuals (82 kg (SD= 8.2), 43 years old (SD=12.5)) without amputation to ensure that the device functioned as intended and to refine the design, if necessary.

## RESULTS

The device fabricated for this study was suitable for able-bodied individuals and included medium-sized thigh and shank shells designed for individuals weighing between 70 and 95 kg. The device does not require customized thigh or shank sections and can be used by able-bodied individuals after size and alignment adjustments have been made. All adjustments can be completed by a clinician or technician, using a 4 mm hex key.

The designed adapter (**Figure 3**) enables a 5 cm shift up or down to accommodate people with different thigh lengths and a 2.5 cm shift to the front or back from the knee joint center, enhancing knee stability and/or function (to provide proper and safe alignment for ambulation). Additionally, this adapter allows clinicians to position the thigh at four different flexion angles (0, 5, 10, 15 degrees) to adjust for a hip flexion contracture. This adapter was made of aluminum flat bar and weighed 57 grams. The overall device weight for the non-amputee version was 3,750 grams.

SolidWorks simulations showed that the device could tolerate loads more than 200 kg. All five testing participants successfully walked with the non-amputee version of the temporary prosthesis prototype, and the device withstood walking, sitting, and standing loads. The weight-bearing load is primarily supported by the thigh section while partially utilizing the patellar tendon and medial/lateral tibial flares. There was no weight-bearing on the foot that was suspended inside the temporary prosthesis. This device ensured that only the left leg and contralateral prosthetic foot contacted the ground. This setup provided 10 centimetres of clearance between the floor and the right foot.

**Figure 5:**
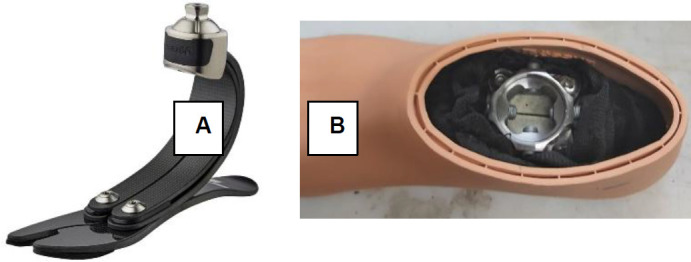
**A:** Pro-Flex^®^ XC foot; **B:** modified Pro-Flex^®^ XC foot with a different adapter.

The shank length is adjustable based on the individual’s leg length. Additionally, different prosthetic feet can be used with this prototype. In this pilot study with able-bodied individuals, the Pro-Flex^®^ XC foot was modified, (https://www.ossur.com/en-ca/prosthetics/feet/pro-flex-xc), creating a very short-profile prosthetic foot to avoid adding unnecessary length to the temporary prosthesis (**Figure 5**). The modified foot was intended solely for internal use with able-bodied individuals within the research and was solely used within a prosthetic facility or a rehabilitation center (i.e., no outdoor evaluations were conducted). For testing the device on people with transtibial amputation, a shorter version of the device will be fabricated that does not require any changes to the prosthetic foot.

## DISCUSSION

In this project, an adjustable temporary training prosthesis was successfully designed, and pilot tested by able-bodied individuals within a prosthetic facility or a rehabilitation centre, with no outdoor evaluations conducted. The device was easily adjusted and aligned to the participant and all participants successfully walked, stood, and sat with the prototype temporary prosthesis.

To enable use by able-bodied individuals, a longer version of the device was developed. The device did not require customized thigh or shank sections. This permitted repetitive evaluations of temporary prosthesis performance. Success with this testing device ensured that future evaluations for people with amputations are appropriate (i.e., device is ready for clinical evaluation).

After undergoing several modifications and iterations, the final prototype device met the design criteria. The designed adapter to connect the thigh section to the uprights, allowed for 2.5 cm front/back shifting, aiding alignment adjustment. Additionally, it accommodated varying thigh lengths with a 5 cm up or down movement capability and provided flexion adjustment options (0, 5, 10, 15 degrees) to address hip flexion contracture. Two off-the-shelf locking knee joints (Modified Ring Lock Knee Joint) used in this prototype, enabled unrestricted knee motion, crucial for smooth ambulation, and featured a safety locking mechanism for instances of instability.

Participants could relieve pressure on the shank section by loosening the shank straps to bear weight more or solely on the thigh section. Furthermore, after instruction and practice, they donned and doffed the device within the designated 3-minute timeframe. Fit and height adjustments were also straightforward, requiring only a 4 mm hex key and approximately 10–15 minutes. The device weighed less than 4 kg, even in its longer version for able-bodied individuals. Device weight could potentially be further reduced in shorter versions, ensuring practicality and user comfort.

To prevent pressure on the suture line in a newly amputated residual limb, weight-bearing in this device primarily occurs through the thigh section, with partial support from the patellar tendon and medial/lateral tibia flares. Based on the design principles and observations during the testing phase, where individuals were able to stand using only the thigh section, it is believed that most of the patient's weight can be tolerated by the thigh section. However, there is a need for further quantitative analysis to measure the forces applied to both the thigh and shank sections for a more comprehensive understanding. In this study, participants were able to adjust pressure or weight-bearing on the thigh or shank sections by tightening or loosening the straps. Evaluation with able-bodied individuals showed that this device could completely offload the foot inside the prosthesis. Potentially, this non-amputee version could be used as an off-loading orthosis in case of ankle or foot fractures.

Compared to the Pneumatic Post-Amputation Mobility Aid (PPAM) and immediate post-operative rigid dressing, this device does not restrict knee movement, which is crucial in rehabilitation.^8^

Training with the temporary prosthesis is necessary, similar to when a patient receives their first prosthesis. After fitting by a prosthetist, a physiotherapist needs to work with the patient on gait training inside the parallel bars. For safety reasons, the rehabilitation team must teach the patient how to use the device, how to adjust the weight bearing on the thigh or shank, and how to walk before they can use the temporary prosthesis outside the parallel bars.

In this pilot study, the temporary training prosthesis was evaluated on five male able-bodied individuals to ensure the device’s safety and functionality. Additionally, the device was solely used within a prosthetic facility or a rehab center (i.e., no outdoor evaluations). Qualitative feedback was the basis for device evaluation.

## CONCLUSION

Future research will involve testing this temporary prosthesis with five experienced prosthesis users who can provide experienced feedback on device fit and function. Subsequently, a larger cohort of individuals with new transtibial amputations will be recruited to verify appropriate function in practice. This device is believed to have the potential to enhance the rehabilitation of individuals with transtibial amputation and could also serve as an off-loading orthosis for those with ankle or shank fractures.

## DECLARATION OF CONFLICTING INTERESTS

The authors declare that they have no conflicts of interest to report.

## AUTHORS CONTRIBUTION

**Hossein Gholizadeh:**

Conceptualization, design and fabrication of the prototypes, data collection and analysis, writing the manuscript, revising the manuscript, final manuscript approval, and ethics certification application.

**Natalie Baddour:**

Conceptualization, supervision, methodology, reviewing/revising the manuscript, final manuscript approval.

**Nancy Dudek:**

Conceptualization, reviewing/revising the manuscript, final manuscript approval.

**Edward D. Lemaire:**

Conceptualization, supervision, methodology, reviewing/revising the manuscript, final manuscript approval.

## SOURCES OF SUPPORT

This project was made possible due to the generous support from The War Amps.
